# Counting Mutagenized Genomes and Optimizing Genetic Screens in *Caenorhabditis elegans*

**DOI:** 10.1371/journal.pone.0001117

**Published:** 2007-11-07

**Authors:** Shai Shaham

**Affiliations:** Laboratory of Developmental Genetics, The Rockefeller University, New York, New York, United States of America; Texas A&M University, United States of America

## Abstract

In genetic screens, the number of mutagenized gametes examined is an important parameter for evaluating screen progress, the number of genes of a given mutable phenotype, gene size, cost, and labor. Since genetic screens often entail examination of thousands or tens of thousands of animals, strategies for optimizing genetics screens are important for minimizing effort while maximizing the number of mutagenized gametes examined. To date, such strategies have not been described for genetic screens in the nematode *Caenorhabditis elegans*. Here we review general principles of genetic screens in *C. elegans*, and use a modified binomial strategy to obtain a general expression for the number of mutagenized gametes examined in a genetic screen. We use this expression to calculate optimal screening parameters for a large range of genetic screen types. In addition, we developed a simple online genetic-screen-optimization tool that can be used independently of this paper. Our results demonstrate that choosing the optimal *F2*-to-*F1* screening ratio can significantly improve screen efficiency.

## Introduction

The identification of mutant animals is usually the first step in the genetic analysis of a biological process. In animals amenable to genetic study, such mutants are commonly identified by random mutagenesis, followed by screening for the trait under study. The mutation frequencies conferred by different mutagens in different organisms have been extensively documented [1]. Furthermore, for a given mutagen dose, the probability of identifying a mutant in a specific gene is a function of the number of mutagenized gametes (or haploid genomes) examined. Typically, mutagens are employed at doses inducing 10–100 mutations per haploid genome. At such mutagenesis frequencies the number of induced mutations is orders of magnitude above the accumulation of spontaneous mutations, yet is not too large as to preclude organismal viability [1].

For screens in the nematode *Caenorhabditis elegans*, typical mutagenesis regimens require isolation and examination of thousands of animals to approach saturation. Depending on the screening approach, examination of such numbers of animals can be quite labor intensive. In these situations, therefore, it is advantageous to identify optimal screening strategies that maximize the number of genomes screened, while minimizing the work involved. Indeed, as we show here, choosing a reasonable, yet suboptimal ratio of *F*2 to *F*1 animals, can double or triple the work involved in screening a given number of mutagenized *F*1 animals, as compared to screening using optimal parameters. Thus, suboptimal screen strategies may unnecessarily prolong screens, and use up excess reagents.

Currently, a description of how to optimize genetic screens in *C. elegans* is not available. We therefore set out to develop a general algorithm for optimizing genetic screens in this organism. Here we examine such an optimization approach. We begin by reviewing the variables affecting the number of mutagenized haploid genomes needed to achieve saturation screening. We then derive a general expression, valid for most genetic screening approaches used in *C. elegans*, for counting the number of mutagenized *F*1 animals examined in a genetic screen. The expression we derive is, as we show, essentially independent of the total number of animals scored during the course of the screen, and is independent of variations in locus mutability. Although the number of mutagenized *F*1 animals is often approximated by the Poisson distribution, we demonstrate that for at least one major screen class, the Poisson approximation leads to large errors.

Using the generalized expression described above, we solve optimization equations to maximize the efficiencies of two common genetic screen types. Our solutions reveal that an optimal *F*2-to-*F*1 screening ratio always exists for these screens, and that this ratio is dependent neither on the total number of animals scored, nor on the number of mutagenized gametes examined. Rather, the optimal screening ratio depends only on the type of mutation sought (*e.g.* recessive, dominant) and on the relative work involved in picking and scoring *F*1 and *F*2 animals. We use our results to delineate a simple algorithm for setting up and following the progress of genetic screens in *C. elegans*.

Although valid for *C. elegans*, our results can be simply extended to genetic screens in other organisms.

## Results

(Readers not interested in the mathematical exposition that follows can skip to the last section of the Discussion, which describes a simple algorithm for optimizing genetic screens in *C. elegans*).

### The Probability of Identifying a Mutant *F*1 Animal

**Figure 1:**
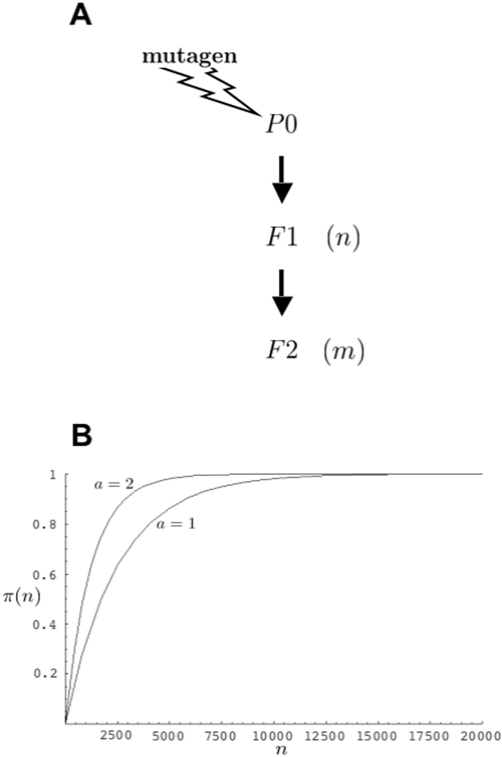
General genetic screening scheme in *C. elegans*. (A) *P*0 animals are mutagenized, and allowed to self-fertilize to produce *F*1 animals. To identify recessive mutations, *F*1 animals are allowed to self-fertilize to produce the *F*2 generation. In this paper we consider the case of *n F*1 animals giving rise to *m F*2 animals. (B) Plots describing the probability, π(*n*), that among *n F*1 animals screened, following mutagenesis by EMS (*r* = 1,250), will be found at least one *F*1 animal heterozygous for a loss-of-function mutation in a gene of interest. The parameter *a* is as defined in the text. The plots were generated by the program Mathematica 5.0 (Wolfram Research), using *n* as a continuous variable.

The nematode *C. elegans* is a self-fertilizing hermaphrodite that, at least under laboratory settings, rarely uses males for reproduction [2]. Genetic screens in *C. elegans* generally follow a scheme similar to the one outlined in Figure 1A. First, animals (the *P*0 generation) are exposed to a mutagen inducing mutations in sperm and oocytes [1]. The mutagen ethyl methanesulfonate (EMS), for example, when used at a concentration of 50 mM, induces loss-of-function mutations in a given *C. elegans* gene at an average frequency of one every 2,500 mutagenized *P*0 gametes [1]–[6]. Generally, fourth-larval-stage (L4) or young-adult animals are used as mutagenesis targets to maximize the probability that the mutations generated are derived from independent events [1].

Next, *P*0 animals are allowed to self-fertilize, to produce *F*1 progeny. If mutations in either copy of a gene under consideration can be revealed in subsequent analysis, and if *n F*1 animals are examined, then the number of haploid genomes screened, defined as the number of *P*0 gametes examined, is given by 2*n*. More generally, however, the number of haploid genomes examined is given by *a* times *n* (*an*) where *a* takes on the value of either 1 or 2. For example, if a mutation in a gene of interest is suspected to be lethal or sterile, attempts to induce it on a marked chromosome, opposite a balancer chromosome, are often undertaken [7]. For such a screen, only mutations induced on the marked, non-balancer chromosome are sought, and *a* = 1.

Following mutagenesis, the probability of finding at least one *F*1 animal heterozygous for a mutation in a specific gene of interest is influenced by a number of parameters, and can often vary greatly from gene to gene. Thus, for example, smaller genes may be less likely to be hit by mutagen. Furthermore, some mutagens, such as EMS, preferentially alter certain nucleotides [8]–[11], thus nucleotide content of a gene may also affect its mutation frequency. It is also possible that chromatin structure and packing of DNA in the environs of a gene may play a role in its mutagenesis frequency. Thus, obtaining an exact estimate for the number of *F*1 animals to be screened is difficult, and screens are generally considered near saturation when multiple alleles of a given gene have been identified.

Nonetheless, in many instances, assuming an average mutagenesis frequency can lead to useful estimates regarding screen progress, and deviations from these estimates can often hint at unique features of a gene, such as unusually large or small size [1], [12], [13]. To calculate such average probabilities we can proceed as follows.

The probability of finding at least one *F*1 animal heterozygous for a mutation in a gene of interest among *n F*1 progeny of mutagenized *P*0 animals, assuming *a* = 2, is 1−(probability no *F*1 animals carry a mutation)−(probability an *F*1 animal carries independent mutations in each copy of a gene of interest). For common mutagenesis frequencies, the last term is exceedingly small and can be ignored without significant loss of accuracy. Thus, the probability of finding a heterozygous *F*1 animal, π(*n*), is given by
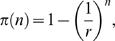
(1)where 1/r is the fraction of *F*1 animals expected, on average, to carry a mutation in a gene of interest. As described above, *r* = 1,250 for loss-of-function mutations obtained by EMS mutagenesis.

Using equation (1), we can calculate how many *F*1 animals should be examined, on average, to obtain at least one animal carrying a loss-of-function mutation in a gene of interest. For example, to achieve a 95% probability (π(*n*) = 0.95) of obtaining an *F*1 carrier, we subtract 1 from both sides of equation (1), divide both sides by −1, take the logarithm of both sides of the equation, and rearrange to obtain n = ln0.05/ln(1−1/r). For large values of *r*, as is the case for most mutagenesis regimes, ln(1−1/*r*)≈−1/*r*. We thus obtain the expression *n*/*r* = −ln0.05, or *n*/*r*≈3, a commonly used result (see also Materials and Methods section of ref. 14).

A similar calculation for *a* = 1 (see example above) is more complex if the *F*1 animals, in which the mutation of interest is induced on the balancer chromosome, cannot be readily distinguished from those in which the mutation is induced on the marked chromosome, and we proceed as follows. Of a collection of *n F*1 animals, assume that *q* are heterozygous for mutations in a given gene. The odds that this is the case are given by the probability of obtaining *q* heterozygotes, (1/*r*)*^q^*, multiplied by the probability of the remaining *n*−*q F*1 animals being non-carriers, (1−1/*r*)*^n−q^*, multiplied by the number of ways such an arrangement can occur, given by the binomial coefficient 
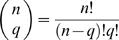
. The probability that at least one of the *q* animals carries the mutation on the marked chromosome is given by 1−(1/2)*^q^*, where (1/2)*^q^* is the probability that all *q* heterozygotes carry the mutation on the balancer chromosome. Thus, the probability that of *n F*1 animals, *q* are heterozygous for mutations in a given gene, and at least one of these *q* animals carries the mutation on the marked chromosome is 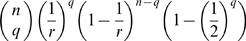. Therefore, to obtain the probability that at least one informative *F*1 animal is present among the *n F*1 animals picked, we sum the individual probabilities for each value of *q* to obtain
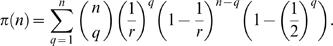
(2)


For large *n* and *r*, equation (2) can be accurately approximated using the Poisson distribution (see Materials and Methods), yielding:
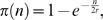
(3)


For a given *n* it is expected that π*_a_*_ = 2_(*n*)> π*_a_*_ = 1_(*n*), a result illustrated in Figure 1B where the probabilities π(*n*) are plotted for values of *n* between 1 and 20,000, assuming *r* = 1,250.

### Counting F1 Animals Screened- an Example

The discussion of the previous section suggests that gene-to-gene variations in mutability make it difficult to predict precisely how many mutagenized *F*1 animals must be sifted through to identify a mutant of choice. However, the question arises as to whether an optimal screening strategy, *independent* of locus mutability, might exist, and if so, what are its properties.

To begin to address this issue, we must first determine how many mutagenized *F*1 animals have been examined during a genetic screen. In this section and the following sections we make a distinction between the number of *F*1 animals *picked* for analysis, *n*, and the number of *F*1 animals whose mutation content has actually been *examined*, *n_act_*. If the mutation being sought is predicted to behave in a simple dominant fashion, then counting how many *F*1 animals were examined is trivial, and is precisely equal to the number of *F*1 animals picked, that is, *n_act_* = *n* (*e.g.* Table 1). However, in most schemes, as illustrated in Figure 1A, recessive mutations are sought, whose presence cannot be discerned in the *F*1 generation. Thus, *F*1 animals are allowed to self-fertilize, to produce *F*2 animals, among which may be identified homozygous mutants in the gene of interest, displaying a scorable phenotype. In this case it is generally the case that *n_act_*≠*n*, and *n_act_* must be used instead of *n* in equations (1), (2), and (3) to obtain π(*n*).

**Table 1:** Optimal Numbers of F1 and F2 Animals Required to Screen 5,000 Mutagenized F1 Animals for Different Screen Parameters.

Screen type	Screen parameters	F1 plating method^a^	No. F1s (n)	No. F2s (m)	Work (W)	Examples of types of mutants sought
F1 screen	N/A	N/A	5,000	0	Depends on ease of scoring phenotype	1. Dominant visible mutants.
						2. Non-complementation screen for recessive mutants.
						3. Intragenic suppression of a dominant mutation.
Ia	α = 1.01^b^	1 F1/plate	11,429	22,857	34,514	1. Male visible mutants where mating must be avoided during the screen.
	γ = 1^c^					2. Maternal-effect sterile mutants.
Ib	α = 0.01	Small number of plates	25,738	22,238	248	1. Visible mutants.
	γ = 0.001^d^					2. Selections (α,γ very small in this case)^e^.
II	α = 0.01	1 F1/plate	5,164	61,963	5,783	1. Sterile mutants.
	γ = 1					2. Lethal mutants.
III	α = 1.01	Small number of plates	224,732	20,226	20,653	1. Maternal-effect visible mutants.
	γ = 0.001					2. Mutants enhancing a weakly penetrant phenotype.
						3. Mutants affecting population behavior.
						4. Male sterile mutants.
						5. Maternally-rescued visible mutants.

All values are for fully penetrant recessive mutations for which p = 0.75. Screen types are defined in the text. N/A, not applicable. All screens are for recessive mutants unless otherwise noted.

a F1 animals are either individually plated at 1 F1 per plate, or plated in bulk (*e.g.* from a liquid culture) on one or a small number of plates.

b α = 1.01, the amount of work to pick 1 F2 animal to a plate is, by definition, equal to 1; it is estimated that to score a visible mutant requires about 1/100 the amount of work of picking 1 F2 animal to a plate (α = 0.01), thus, α is the sum of these.

c γ = 1 is, by definition, the work to pick one F1 animal to a plate using a worm pick.

d γ = 0.001, it is estimated that the amount of work to bulk plate 1 F1 animal is equal to 1/1000 the amount of work to pick one F1 animal using a worm pick.

e In this case the values of α and γ essentially approach 0.

To obtain a general expression for *n_act_*, we begin by considering, as an example, a genetic screen in which all *n F*1 animals are picked to a single plate, from which *m F*2 animals are then scored for the mutant phenotype. We assume that all *F*1 animals produce approximately the same number of *F*2 animals. Indeed, for standard EMS screens in *C. elegans*, only about 5% of *F*1 animals have greatly reduced fertility, and fewer than 1% of *F*1 animals have reduced fertility using one common protocol for trimethylpsoralen and ultraviolet light mutagenesis (S.S., unpublished results). Furthermore, for many mutagenesis schemes, only healthy *F*1 animals are picked for subsequent screening, further reducing the number of animals producing low brood counts.

For such a screen design, *n_act_* can be approximated fairly accurately in two limiting cases. First, we consider a screen in which *m*≫*n*. In this case, many *F*2 progeny have been scored for each *F*1 animal, making it very likely that the mutation content of all *F*1 animals has been established. Therefore, *n_act_*≈*n*.

Second, we consider the case for which *n*≫*m*. Here, the likelihood that two or more of the *F*2 animals scored derive from the same *F*1 animal is small. Thus, *n_act_*≈*m*(1−*p*), where *p*, is the probability that a scored *F*2 has failed to reveal whether its *F*1 parent had the mutation of interest. In the case of simple screening schemes involving recessive mutations, *p* = 3/4.

Although these limiting cases have important uses, genetic screens that involve manually picking *F*1 animals, *F*2 animals, or both, may fail to satisfy the limiting conditions considered above. To calculate *n_act_* for the general case of this example, we proceed as follows.

The probability that among the *m* scored *F*2 animals are represented *q* progeny of a particular *F*1 animal is given by the binomial term
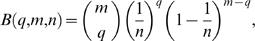
(4)where (1/*n*)*^q^* is the probability of scoring *q* progeny of a particular *F*1 animal, (1−1/*n*)*^m−q^* is the probability of the remaining scored *F*2 animals not being progeny of the particular *F*1 animal under consideration, and 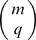 representing the number of ways such a combination can be picked.

The probability that at least one of the *q* scored *F*2 animals is informative about the presence of a mutation of interest in the particular *F*1 animal under consideration, is given by 1−*p^q^*, where *p* is the probability of the *F*2 animal not being informative. Therefore, by analogy to the calculation in the previous section, the probability that at least one informative *F*2 progeny of a particular *F*1 animal is found among the *m F*2 animals scored is given by
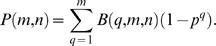
(5)


In this paper we aim to describe optimal genetic screening strategies, and are, in general, interested in the optimal screening ratio of *F*2 to *F*1 animals, *y* = *m*/*n*. It will become useful, therefore, to represent the functions *B*(*q*,*m*,*n*) and *P*(*m*,*n*) as functions of *y* and *N* = *n*+*m*, the total number of animals picked in the screen. By making the appropriate substitutions we can rewrite these functions as
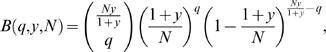
(6)


and
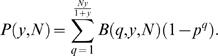
(7)The number of *F*1 animals examined for possession of a mutation in a specific gene, then, is given by
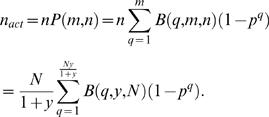
(8)


**Figure 2:**
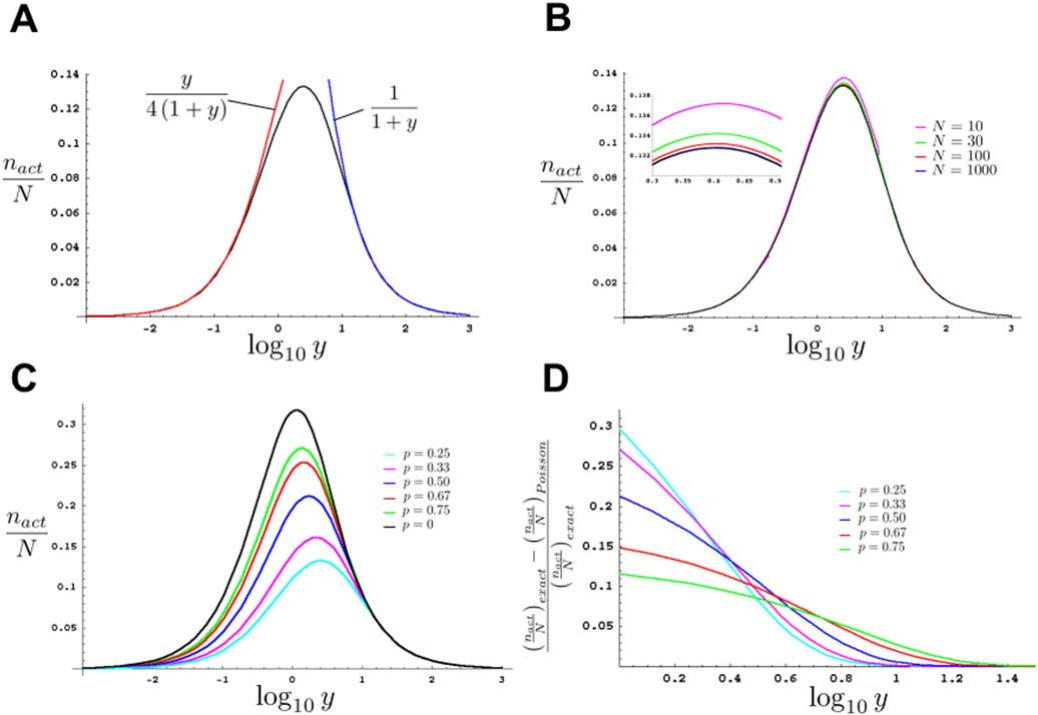
The effects of different parameters on the number of haploid genomes screened. (A–C) Plots assuming all *F*1 animals are placed together on a single plate. *p* = 0.75, unless otherwise indicated. All plots were generated using the program Mathematica 5.0 (Wolfram Research). (A) Black line, graph of *n_act_*/*N vs.* log_10_ *y* for *N* = 1,000. Blue and red lines, graphs of asymptotes and their equations. (B) Graphs of *n_act_*/*N vs.* log_10_ *y*. Different colors indicate *n_act_*/*N* for different specified values of *N*. Black line, Poisson approximation, colored lines, exact solutions. Inset, magnification of the graph for the region of log_10_ *y* between 0.3 and 0.5. For each value of *N*, *y* can only take on values such that 1/(*N*−1)≤*y*≤*N*−1. Furthermore, although for illustration purposes we have drawn the curves as continuous, *y* is *not* a continuous variable, and treating it as such only works for large *N*. This is most obvious for *N* = 10 where *y* can only take on the values 1/9, 1/4, 3/7, 2/3, 1, 3/2, 7/3, 4, and 9. (C) Graphs of *n_act_*/*N vs.* log_10_ *y* for varying values of *p*, as defined in the text. Graphed using the Poisson approximation. (D) Graphs depicting the fractional error incurred when using the Poisson approximation to estimate *n_act_*/*N* for screens in which one *F*1 animal is plated per plate. Although graphs are continuous, only integer values of *y* are relevant. Also note that the smallest allowable value of *y* is chosen so that at least 1 *F*2 animal is chosen per plate.

In Figure 2A we plot the quantity *n_act_*/*N* as a function of log_10_ *y* for a simple recessive mutation with *p* = 0.75 and *N* = 1000. As expected, for *n*≫*m*, *n_act_*/*N* is asymptotic to the curve *m*/(4*N*) = *y*/(4(1+*y*)) (red line). For *m*≫*n*, *n_act_*/*N* is asymptotic to the curve *n/N* = 1/(1+*y*) (blue line). As is evident from the figure, for *p* = 0.75, the asymptotic curves overestimate *n_act_* by only 5% or less for *n* and *m* such that *y* = *m*/*n*≤0.4 or *m/n*≥12.2. However, in between these values, a very large error, that may exceed 100% of the true value of *n_act_*, can occur (not shown), justifying the more detailed analysis presented in this section. For large *m* and *n*, equations (7) and (8) are very closely approximated using the Poisson distribution (see Materials and Methods) to obtain
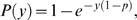
(9)


and
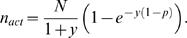
(10)


Figure 2B demonstrates this graphically, showing the tight agreement between equation (8) and the Poisson approximated equation (10) (black line), for values of *N* as small as 10.

Figure 2B also demonstrates the advantage of using the scaled parameter *n_act_*/*N* in our analysis. Although equation (8) shows that *n_act_*/*N* is dependent on the value of *N*, Figure 2B reveals that this dependence is very weak. Indeed, equation (10) shows that for large *m* and *n*, *n_act_*/*N* becomes entirely independent of *N*, making *n_act_*/*N* useful for the analysis of most screens.

### Single-Plate Screens for Mutants That Are Not Strictly Recessive

Although fully penetrant recessive mutations for which *p* = 0.75 are the most commonly sought mutations [15], other screening modes are frequently used for which *p*≠0.75. For example, if the *F*1 animals are heterozygous for a balancer chromosome and an *un*marked homologous chromosome, such that animals homozygous for the balancer are dead, or easily identifiable, then *p* becomes 2/3 = 0.67 for a fully penetrant recessive mutation. As another example, dominant mutations that are rescued by a wild-type maternal genotype cannot be isolated in the *F*1 generation, but can be sought among *F*2 animals. For strictly dominant mutations of this type, *p* = 0.25.

The case *p* = 0 occurs when *F*1 animals are heterozygous for a balancer and a marked chromosome, with the mutation of interest induced on the latter. In this case, only marked *F*2 animals are scored, and these should all be homozygous for the mutation of interest. The condition *p* = 0 also holds for rare screens where dominant maternal-effect mutants are sought. In such screens, heterozygous *F*1 animals do not have the phenotype of interest, but all of their progeny do.

The condition *p* = 0 arises frequently in genetic screens of haploid organisms. In this case, *n_act_*/*N* is a measure of the representation of an initial pool of mutagenized organisms in the progeny that have been examined.

In Figure 2C we plot curves of *n_act_*/*N v*s. log_10_ *y* for a number of possible values of *p*. The curves agree with the expectation that the more informative an *F*2 animal is about the mutation state of its *F*1 parent (*i.e.*, the smaller *p* is), the fewer *F*2 animals must be examined to achieve a specific value of *n_act_*/*N*.

### The General Expression for the Number of *F*1 Animals Screened

The analysis described in the preceding two sections holds for the specific case in which all *F*1 animals are placed on a single plate, from which *F*2 progeny are sampled. However, usually, it is necessary for *F*1 animals to be placed individually, or in groups, on multiple plates. *F*2 animals are then drawn from each plate. Individual plating of *F*1 animals is a particularly common scheme that is utilized if the expected mutation is lethal, and heterozygous *F*2 siblings need to be recovered, or in a situation where males are present in the population and mating between *F*1 animals must be avoided. Should *F*1 plating strategy affect *n_act_*? Consider the case where single *F*1 animals are placed on individual plates, and equal numbers of *F*2 progeny are drawn from each plate. Because we know for certain that in such a scheme *F*2 animals scored are derived from every *F*1 animal picked, it is predicted that for a given *y*, *n_act_* should be greater than in a scheme in which all *F*1 animals were plated on a single plate, where some *F*1 animals may not be sampled in the *F*2 generation. Thus, for the same number of *F*1 and *F*2 animals, *n_act_* will indeed be dependent on plating strategy.

To calculate the general expression for *n_act_* explicitly, we let ν equal the number of *F*1 animals picked to a plate, and μ equal the number of *F*2 animals scored per plate. In general, ν and μ can be different for every plate. 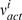, the actual number of *F*1 animals examined on the *i*th plate, is given, as in equation (8), by
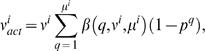
(11)where
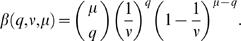
(12)The general expression for *n_act_*/*N*, is, therefore, obtained by summing the actual number of *F*1 animals examined over all plates, or
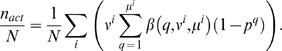
(13)


Equation (13) is valid for essentially every type of genetic screen, involving any plating strategy. The equation can be significantly simplified if we assume that the same number of *F*1 and *F*2 animals are plated and scored per plate. In this case, ν*^i^*, μ*^i^*, and β(*q*, ν*^i^*, μ*^i^*) are identical for each plate, and equation (13) can be written as
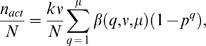
(14)where *k* is the number of plates examined.

Equation (14) can be expressed in terms of *y* as follows. To express μ as a function of *y* we note that *y* = *m*/*n* = *k*μ/(*k*ν) = μ/ν. Rearranging terms yields μ = ν*y*. Also, *k*ν/*N* = *n*/(*n*+*m*) = 1/(1+*m*/*n*) = 1/(1+*y*), yielding
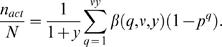
(15)


Four points regarding equation (15) are of note. First, for the common plating strategy of one *F*1 animal/plate, equation (15) can be reduced to
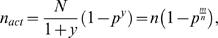
(16)an equation of considerable practical value. Furthermore, as expected, for a single plate with all *F*1 animals on the same plate, ν becomes *n*, and β(*q*,ν,*y*) becomes *B*(*q*,*y*,*N*), as in equation (8).

Second, unlike equations (5) and (8), equation (15), even for large *N*, is *not* well-approximated by the Poisson distribution if a small number of *F*1 animals is plated per plate, which is generally the case for clonal screens. In Figure 2D we plot the error introduced in *n_act_*/*N* by using the Poisson approximation for different values of *p* for the case of one *F*1 animal per plate. Note that for *p* = 0.25, the error can be nearly 30% off the exact value. Indeed, it can be shown that the fractional error, [(*n_act_*/*N*)*_exact_*−(*n_act_*/*N*)*_Poisson_*]/(*n_act_*/*N*)*_exact_*, plotted in Figure 2D approaches a maximal value of *p*−*e* ^(*p*−1)^/(*p*−1) as *y*→1 (see Materials and Methods).

Third, equation (15) reveals that *n_act_*/*N* is independent of *N*, although for a given *N* only values of μ+ν that are divisors of *N* are possible.

Fourth, for a given *N*, the larger the number of *F*1 animals per plate (*k*Ø1), the better is equation (15) approximated by equation (10). Simulations for different ratios of *n* to *k* reveal that for *n/k*≥30 equation 10 gives an excellent estimate of *n_act_* (error<5%; data not shown).

### Optimizing Genetic Screens- Preliminaries

The results described in the previous sections provide us with the appropriate tools to consider how to optimize genetic screens in *C. elegans*. In general, a measure of screen efficiency should take into account the amount of work performed in a screen as well as the total number of mutagenized *F*1 animals examined, *n_act_*. Work can be defined in a number of ways. Here, we will generally define work as the amount of time spent picking and scoring animals. Alternatively, work can be a measure of the total amount of reagents needed for the screen, *etc.*, and much of the analysis that follows would still be valid using this definition. Regardless of the precise definition of work used, the work expended in a screen must be of the form γ*n*+α*m*, where γ and α represent work per animal and have values between 0 and ∞. If we measure work in such a way that γ = α = 1 defines a unit of work, we can write the total work expended as *W* = γ*n*+α*m*. Given this definition, we now propose to define the efficiency of a genetic screen as
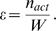
(17)That is, the efficiency is a direct measure of the number of mutagenized *F*1 animals examined per unit of work. As we will demonstrate below, this definition allows us to make quantitative estimates of the optimal screening ratio, *y*, for use in a broad range of genetic screen types.

We note that for screens in which a unit of work is defined as the time spent picking a single animal to a plate, and in which equal work is expended to pick *F*1 and pick and score *F*2 animals, we define γ = α = 1; then, *W* = *N*, and ε = *n_act_*/*N*, which is the parameter we have used throughout this paper (*e.g*. Figure 2A).

We can now pose the optimization problem for genetic screens as follows: what is the *F*2-to-*F*1 screening ratio that minimizes the amount of work needed to screen a required number of mutagenized *F*1 animals, *n_req_*. Before we address the problem, it is worth considering whether the problem is itself a reasonable one. That is, is it reasonable to assume that a minimal value of *W* exists for every genetic screen? An examination of Figure 2A shows that *n_act_*/*N* has a maximum with respect to *y*, suggesting that at least for the specific case of γ = α = 1, an optimal *F*2-to-*F*1 screening ratio, *y_max_*, exists. Extending this result, it can be demonstrated (see next section) that a maximum indeed exists for every value of γ and α>0.

**Figure 3:**
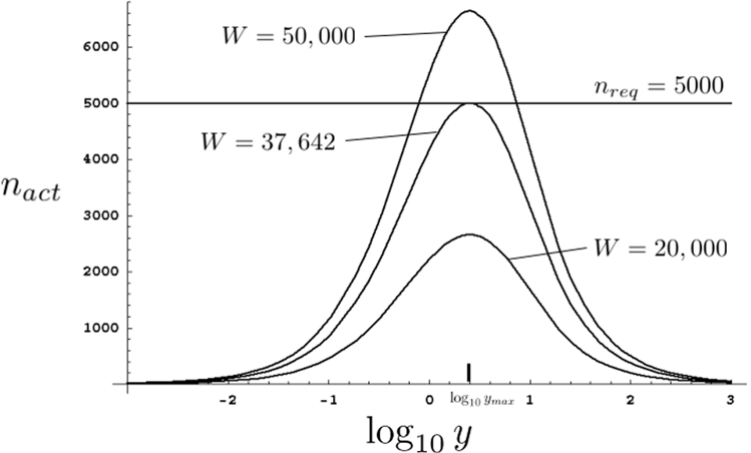
Determining parameters for maximally efficient screens. Graphs of *n_act_ vs.* log_10_ *y* are plotted for three different values of *W*. For *n_req_* = 5,000 (horizontal line), the minimal value of *W* is 37,642. log_10_ *y_max_* is indicated. Plots generated by the program Mathematica 5.0 (Wolfram Research).

Consider Figure 3, where we have plotted *n_act_* as a function of log_10_ *y* for three different values of *W* where γ = α = 1. The minimal amount of work, *W_min_*, required to screen 5,000 mutagenized *F*1 animals, *n_req_* = 5,000, occurs at *W* = 37,642. Values of *W* smaller than this will never achieve *n_req_*, while values of *W* greater than 37,642 do not minimize *W*, by definition. We can therefore formulate the following general criteria: *W_min_* is the value of *W* satisfying the two conditions, 
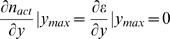
, and *n_act_*(*W_min_*,*y_max_*) = *n_req_*. Although these equations can be numerically solved for the general case represented by equation (13), we restrict our analysis below to the two most common screen cases for which either equations (10) or (16) are valid.

### Optimizing Genetic Screens- All *F*1 Animals on a Single Plate

For genetic screens involving plating all *F*1 animals on a single plate or on a small number of plates relative to the total number of *F*1 animals examined, we can use the Poisson approximation to write the efficiency of a screen as:
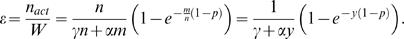
(18)


We differentiate this equation with respect to *y* and set the result equal to 0 to obtain the following transcendental equation for *y_max_*, the optimal *F*2-to-*F*1 screening ratio:
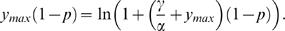
(19)


Two features of this equation are of interest. First, the equation cannot be solved analytically, and must be evaluated numerically. Second, and more importantly, the value of *y_max_* is only dependent on the relative values of γ and α. Thus, the optimal screening ratio, *y_max_*, is independent of the absolute work expended to pick and pick and score *F*1 and *F*2 animals, respectively.

Does *y_max_* always exist? Inspection of equation (19) reveals that it is of the general form *x* = ln(*a*+*x*), where *x* = *y*(1−*p*), and *a* is always greater than 1. Consider the range of possible values of *x*, from 0 to ∞. At *x* = 0, the left hand side of the preceding equation is always smaller than the right hand side (ln*a*). As *x*→∞, ln(*a*+*x*)≈ln(*x*), and thus the right hand side of the equation is always smaller than the left hand side. Since both *x* and ln(*a*+*x*) are continuous functions, these observations mean that there always exists an intersection point of the functions *x* and ln(*a*+*x*), defining *y_max_*. Therefore, *y_max_* exists for all values of α and γ. Differentiation of ε twice with respect to *y* shows that 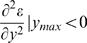 for all values of *y_max_*, guaranteeing that *y_max_* indeed represent a global maximum of ε.

**Figure 4:**
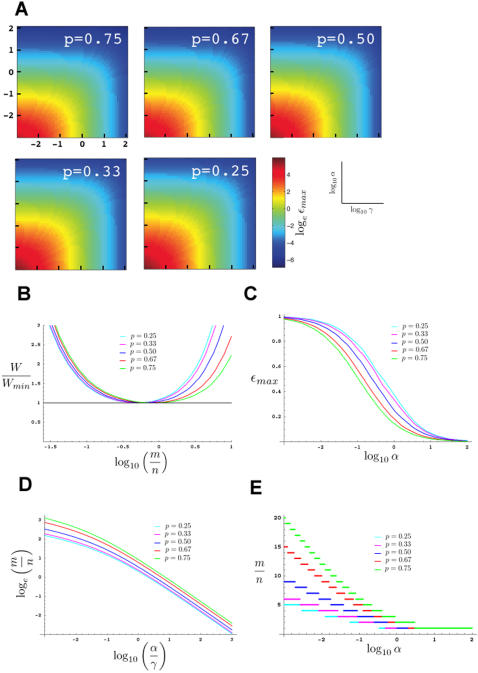
Optimal *F*2-to-*F*1 screening ratios and screen efficiency calculations. (A) Contour plots examining maximal screen efficiency, ε*_max_*, as a function of α and γ, for different values of *p*, for screens where all *F*1 animals are plated on one or a small number of plates. Plots generated using the program MatLab (MathWorks). (B) Graphs examining fold increase in work performed as screening ratio (*m*/*n*) deviates from its optimal value, for screens where all *F*1 animals are plated on one or a small number of plates and α/γ = 10. (C) Graphs depicting maximal screen efficiencies as a function of α for screens in which *F*1 animals are plated individually. In these graphs γ = 1, which is the most common value for this screening mode. (D) Graphs of the optimal *F*2-to-*F*1 screening ratios (*m*/*n*) for different values of *p* as functions of α/γ, for screens where all *F*1 animals are plated on one or a small number of plates. Note that the vertical axis is the natural log of *m*/*n* and not the base 10 log. (E) Graphs of the optimal *F*2-to-*F*1 screening ratios (*m*/*n*) for different values of *p* as functions of α, for screens in which *F*1 animals are plated individually. In these graphs γ = 1, which is the most common value for this screening mode.

Figure 4D depicts values of the optimal screening ratio for a range of ratios of α and γ. Insertion of *y_max_* into equation (18) yields ε*_max_* for given values of α and γ. Figure 4A depicts ε*_max_* for a range of values of α and γ and for different values of *p*. As expected, the smaller the values of α and γ, the larger is ε, and the more efficient the screen.

### Optimizing Genetic Screens- One *F*1 Animal per Plate

For genetic screens involving plating one *F*1 animal per plate, the Poisson approximation cannot be used (see above), and instead we use equation (16) to write the efficiency of a screen as:
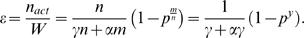
(20)


As above, we differentiate equation (20) to obtain the following transcendental equation for *y_max_*:
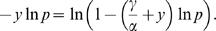
(21)


Similar reasoning to that of the previous section guarantees the existence of a solution for *y*. However, the value of *y* obtained here cannot be used directly to compute *y_max_* or to calculate ε*_max_*, for two reasons. First, unlike the previous section, *y* cannot be treated as a continuous variable here, and the maximum calculated in equation (21) makes this assumption. Second, *y* can only take on integer values ≥1. Thus, to identify *y_max_*, we numerically calculate the solution to equation (21). We then check whether the obtained value of *y* is smaller than 1. If so, then *y_max_* = 1. If not, we calculate the efficiency of screening, using equation (20) for the two nearest integer values of *y*, and choose *y_max_* as the value giving the highest value of ε. The results of these calculations for ε*_max_* and *y_max_* are presented in Figures 4C and 4E, respectively. Note that in these figures we have assumed γ = 1, since this is most often the case when performing a screen of the type considered here. However, other values of γ may be possible, in which case α in this figure should be replaced by α/γ.

### Deviations from Maximal Screening Efficiency

In Figure 4B we plot the ratio of the work expended in a genetic screen, *W*, to the minimal work, *W_min_*, calculated using equations (17), (18), and (19), as a function of *m*/*n*, for a screen in which scoring and picking *F*2 animals requires ten times more work than picking *F*1 animals (α/γ = 10). Such parameters are often encountered, and may serve as a model for screens in which *F*2 animals need to be examined individually on a compound microscope, for example. As the figure demonstrates, relatively small deviations in the screening ratio can have a large impact on the amount of work carried out to screen the same number of mutagenized *F*1 animals. For example, screening five *F*2 animals for every *F*1 parent for recessive mutations nearly doubles the work required to screen the same number of mutagneized *F*1 animals compared to the optimal screening ratio of 0.86. The differences are even more dramatic when dominant mutations are sought.

These results clearly show that using optimized screen parameters can have a significant impact on the progress and output of genetic screens in *C. elegans*.

## Discussion

### Summary of Key Points

In this paper, we derive a strategy for optimizing genetic screens in the nematode *C. elegans*. We demonstrate two key points. First, an optimal screening strategy always exists for every genetic screen of the types considered here. Second, calculation of this optimal strategy is possible. Figures 4D and 4E depict the results of such calculations, displaying the optimal *F*2-to-*F*1 screening ratios for a large range of screen parameters. As shown in Figure 4B, using optimal screening parameters for screens of any type can make a significant difference in the amount of time, labor, and/or reagents used to identify mutants of interest. This difference in efficiency between optimal and suboptimal screening strategies is most accentuated under two condition: when there is a significant difference in the work done picking *F*1 animals and picking and scoring *F*2 animals; and when the mutations sought manifest themselves in the *F*2 population in increased proportion (as might occur with a dominant mutation; Figure 4B).

Our results suggest a general rule of thumb: in pursuing a genetic screen, optimal efficiency is achieved by minimizing as much as possible the more difficult task between picking *F*1 animals or picking and scoring *F*2 animals.

In addition to the key results discussed above, we have also derived a number of other useful results. First, we have shown that the optimal screening strategy does not depend on the total amount of effort expended in a screen, but only depends on the ratio of the work involved in picking *F*1 animals to the work expended in picking and scoring *F*2 animals, and on the type of mutation being sought. Second, our studies reveal that use of the Poisson approximation to count the number of mutagenized *F*1 animals examined in a screen is not appropriate for all situations. Third, instead, we derive an equation (equation (13)), that is valid for a large number of genetic screens. Fourth, we demonstrate that two limiting cases of this equation, in which a large number of *F*1 animals are placed on a small number of plates, or in which *F*1 animals are plated individually, yield simplified equations (equation (10), based on the Poisson approximation, and equation (16)), that are well known and of considerable practical use. Finally, analysis of these limiting cases also reveals that the number of mutagenized *F*1 animals examined is dependent on the mode in which *F*1 animals are plated. In general, we show that plating *F*1 animals individually, followed by scoring their *F*2 progeny, allows more mutagenized *F*1 animals to be examined than plating the same number of *F*1 animals on a single or small number of plates. However, it should be noted that because plating *F*1 animals individually can be more time consuming and may require more reagents, the overall screen efficiency may or may not be higher using this strategy (see below).

### A Classification of Genetic Screens

**Figure 5:**
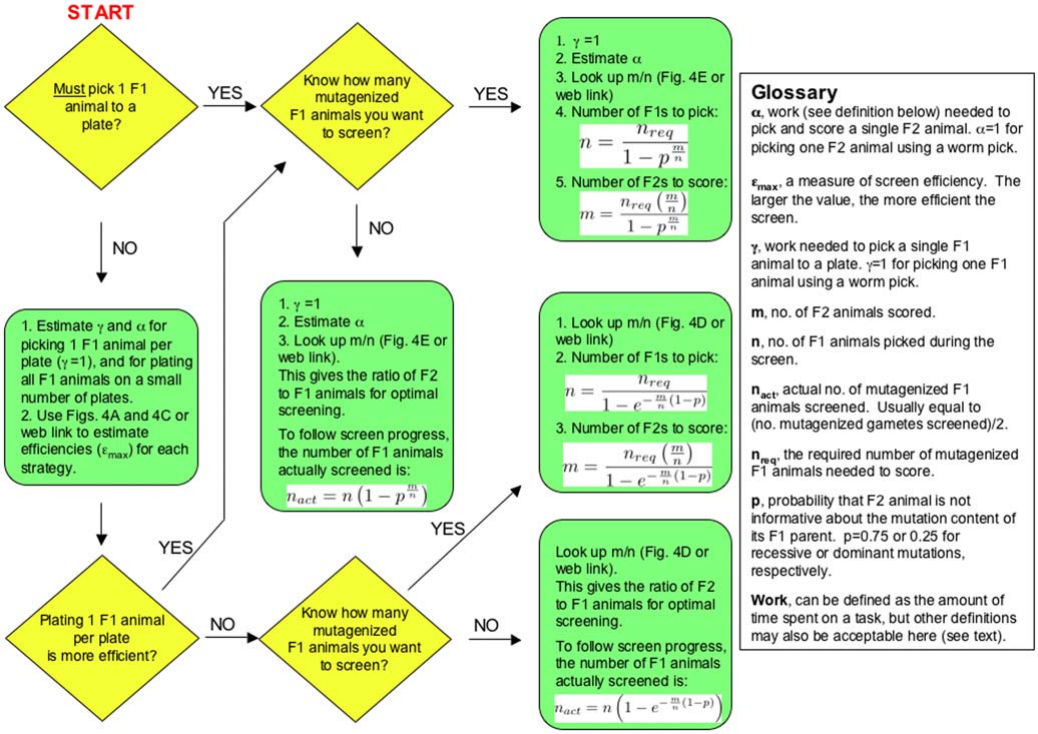
Algorithm for performing an optimal genetic screen. Flowchart begins on the top left corner at “START”. All parameters and equations are described and derived in the text. Parameters of relevance are also described in the Glossary portion of the figure. Diamond shapes indicate steps where a choice must be made.

Our analysis suggests that genetic screens in *C. elegans* can be divided into three general categories, based on the difficulties involved in picking *F*1 animals (the value of γ, see Results and Figure 5) and picking and scoring *F*2 animals (the value of α, see Results and Figure 5).

#### Type I

Picking *F*1 animals and picking and scoring *F*2 animals is of similar magnitude of difficulty. A remarkable consequence of our analysis is that regardless of the type of screen, or the work involved, all screens for which α/γ is fixed, have the same optimal screening ratio. Thus, for screens of this type the ratio of α and γ will determine the precise *F*2-to-*F*1 screening ratio. For screens of this type, equations (10) and (16), can be used to determine *n_act_* and follow screen progress.

#### Type II

Picking *F*1 animals is much harder than picking *F*2 animals (α/γ→0). As shown in Figure 4D, for very small values of α/γ, the optimal screening ratio becomes large, and thus, many *F*2 animals should be scored for each *F*1. As we showed in the beginning section of the paper, under such conditions *n_act_*≈*n*, and screen progress is limited by the number of *F*1 animals that can be examined.

#### Type III

Picking/scoring *F*2 animals is much more difficult than picking *F*1 animals (α/γ→∞). Figure 4D reveals that as α/γ becomes large, the optimal screening ratio becomes small, so that the number of *F*1 animals picked should be much greater than the number of *F*2 animals scored. Under these conditions, *n_act_*≈*m*(1−*p*), and progress is determined by the type of mutation being sought (*e.g.* recessive, dominant), and by the number of *F*2 animals screened.

The merits of the classification system described above are that it allows a quick determination of whether an in-depth analysis of screen parameters is required to follow screen progress. Specifically, for screens of types II and III, calculating screen progress (*n_act_*) is very simple and does not depend on plating strategy. Screens of type I, however, require a more detailed study of the screen and plating parameters.

In Table 1 we compare the optimal screening ratios to screen 5,000 mutagenized *F*1 animals for recessive mutations for different estimated values of α and γ (see table legend for estimation procedure; also see next section). The table illustrates a number of points. First, it provides estimates of the amount of work expended for each screen. Although the table provides exact numbers, it is important to note that these numbers only approximate the actual work involved because some of the parameters used in calculating the work, such as α and γ, may not be exact. Second, it provides specific applications of the different screen strategies to common screens undertaken in *C. elegans*. Third, the table demonstrates that strategies that minimize both α and γ are, as expected, most efficient (see also Figure 4A).

Interestingly, examination of Figure 4A shows that screen efficiency is not perfectly symmetric with respect to α and γ. This lack of symmetry is partially a function of *p* and, it is easily shown that the maximal efficiency, (ε*_max_*), of screens of type II approaches 1/γ, whereas the efficiency of screens of type III approaches (1−*p*)/α. Thus, if one is debating between screening strategies of type II and III where γ(type II)≈α(type III), screens of type II will always be more efficient.

### Estimating α and γ

As we describe in the Results section, precise values of α and γ are not important for calculating the optimal *F*2-to-*F*1 screening ratio. Indeed, as equations (19) and (21) show, only the ratio of α to γ is relevant. It turns out, however, that even an exact measurement of this ratio is not needed in practice. The reason for this is shown in Figure 4D. As shown in this figure, varying α/γ over six orders of magnitude, only changes the *F*2-to-*F*1 screening ratio by two orders of magnitude (note that the vertical axis is the natural log, and not base 10 log of the screening ratio). Thus, the screening ratio is not very sensitive to variations in α and γ. This observation is of clear practical importance, since it is not always easy to precisely compare the work involved in picking *F*1 animals and picking and scoring *F*2 animals. These results suggest that order-of-magnitude estimates of the relative work involved will give good estimates of the appropriate screening ratio.

In Table 1 we provide estimates for α and γ for picking and scoring animals in the course of different genetic screens. The estimates are based on personal experience of the author (see legend to Table 1), and may vary with individual expertise and protocol. However, since the values of α and γ are essentially user defined, individual variations in estimating screen parameters do not affect the results presented here. More accurate values of α and γ can be obtained from pilot screens, which are often carried out anyway, where these parameters can be measured directly by keeping track of the amount of work done (time spent) to pick a fixed number of *F*1 animals and pick and score a fixed number of *F*2 animals. Each work segment is then divided, respectively, by either the number of *F*1 or *F*2 animals to obtain γ and α, respectively. Values for these parameters can also be adjusted as a screen proceeds, based on estimates derived from earlier stages of screening.

### An Algorithm for Optimal Screening

Our results suggest that the design of many types of genetic screens in *C. elegans* can follow a simple set of rules. In Figure 5 we present a simple algorithm for designing a genetic screen and for following screen progress. Initially, a choice must be made as to whether *F*1 animals will be plated onto individual plates (individually) or onto a small number of plates (bulk). This choice is usually not driven by efficiency, but by constraints of the screen. For example, screens that require a clonal strategy, because the identity of the *F*1 parent is important (as might occur in screens for recessive lethals, where heterozygous siblings are to be isolated), demand plating *F*1 animals individually.

If both plating strategies are applicable, the choice of which plating strategy to use will be determined by comparing the efficiencies of each strategy. For example, consider a screen for recessive mutations in which it is not necessary to keep tabs on the *F*1 parents, and for which *F*2 animals must be picked to individual plates for scoring (α≈1; for definitions of α and γ see Glossary of Figure 5, or Results section). Two possible screen options are: *F*1 animals will be plated individually and therefore α≈1 and γ = 1; or, *F*1 animals will be plated in bulk where instead of picking *F*1 animals with a pick, they are loaded from a synchronized liquid culture, and thus γ≈0.001, but α is still about 1. Examination of Figures 4C and 4A, respectively (or using the webcalculator, see below), reveals that the efficiency of the first screen is 0.145, whereas the efficiency of the second screen is 0.245. Therefore, if the screens are carried out at maximal efficiency, the second screen is about 1.7 times more efficient. On the other hand, if in the second screen, animals are plated on a small number of plates, but picked individually to those plates, instead of being loaded from a liquid culture, then γ≈1, and the efficiencies of the first and second screens are 0.145 and 0.133, respectively. Thus, in this case, even though there is no need to preserve the identity of each *F*1 parent, an individual plating strategy is more efficient.

Once plating strategy is established, a determination of whether a fixed number of mutagenized *F*1 animals is to be screened, or whether the screen will be open ended is made. Estimates of α and γ are obtained (if these have not been obtained already), and these are used to identify the optimal *F*2-to-*F*1 screening ratio ( = *m*/*n*) (Figures 4D, and 4E). From this ratio, the indicated equations in Figure 5 may be used to obtain the numbers of *F*1 and *F*2 animals to be screened. If the screen is open ended, progress can be followed using the indicated equations.

To aid with the calculations described here, in Figure 5, and throughout the paper, we have developed a website with a simple interface that can be used independently of this paper. The site yields precise numerical results, and is thus more accurate than Figure 4, where, by necessity, precise values are difficult to read from the graphs. The site can be accessed at “http://b5.rockefeller.edu/cgi-bin/labheads/shaham/genetic_screens/screenfrontpage.cgi”.

### Conclusion

We have presented a systematic approach for optimizing genetic screens in *C. elegans*, and calculated optimal *F*2-to-*F*1 screening ratios for a large range of screen parameters. Calculation of these parameters was aided by obtaining a general expression for counting the number of mutagenized gametes examined in the course of a genetic screen. The strategies described here can, in principle, be applied, with relevant modifications, to the evaluation of equivalent parameters in genetic screens in other organisms.

## Materials and Methods

### Derivation of Poisson approximations

In this paper, we employ expressions of the following form
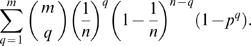
Such expressions can be simplified using Poisson terms to approximate the respective binomial terms. Specifically, it is well known that for large *m* and *n*,
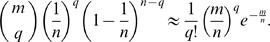
Thus, we can write
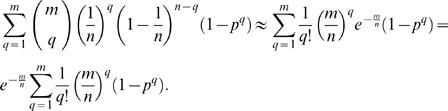
Expanding parentheses, this term can be rewritten as
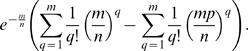
Using the series expansion 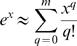 for large *m* and *n*, we simplify the expression above to
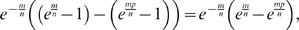
leading to the approximation
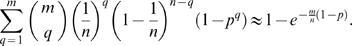



### Derivation of maximal error in using the Poisson approximation when plating *F*1 animals singly

The fractional error in using the Poisson approximation is defined as *f* = [(*n_act_*/*N*)*_exact_*−(*n_act_*/*N*)*_Poisson_*]/(*n_act_*/*N*)*_exact_*. Using equations 10 and 16, the fractional error is written as
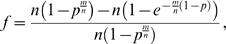
which upon rearrangement yields
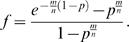
As *y* approaches 1, this expression, therefore becomes
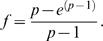


